# LKB1 and Notch Pathways Interact and Control Biliary Morphogenesis

**DOI:** 10.1371/journal.pone.0145400

**Published:** 2015-12-21

**Authors:** Pierre-Alexandre Just, Alexis Poncy, Sara Charawi, Rajae Dahmani, Massiré Traore, Typhanie Dumontet, Valérie Drouet, Florent Dumont, Hélène Gilgenkrantz, Sabine Colnot, Benoit Terris, Cédric Coulouarn, Frédéric Lemaigre, Christine Perret

**Affiliations:** 1 INSERM, U1016, Institut Cochin, F-75014 Paris, France; 2 CNRS, UMR8104, F-75014 Paris, France; 3 Université Paris Descartes, F-75014 Paris, France; 4 Equipe labellisée LNCC Paris, Paris, France; 5 APHP, Hôpitaux Universitaires Paris Centre, Hôpital Cochin, Pathology department, F-75014 Paris, France; 6 de Duve Institute and Université catholique de Louvain, B-1200 Brussels, Belgium; 7 INSERM, UMR991, Université de Rennes 1, F-35033 Rennes, France; Texas A&M Health Science Center, UNITED STATES

## Abstract

**Background:**

LKB1 is an evolutionary conserved kinase implicated in a wide range of cellular functions including inhibition of cell proliferation, regulation of cell polarity and metabolism. When *Lkb1* is inactivated in the liver, glucose homeostasis is perturbed, cellular polarity is affected and cholestasis develops. Cholestasis occurs as a result from deficient bile duct development, yet how LKB1 impacts on biliary morphogenesis is unknown.

**Methodology/Principal Findings:**

We characterized the phenotype of mice in which deletion of the *Lkb1* gene has been specifically targeted to the hepatoblasts. Our results confirmed that lack of LKB1 in the liver results in bile duct paucity leading to cholestasis. Immunostaining analysis at a prenatal stage showed that LKB1 is not required for differentiation of hepatoblasts to cholangiocyte precursors but promotes maturation of the primitive ductal structures to mature bile ducts. This phenotype is similar to that obtained upon inactivation of Notch signaling in the liver. We tested the hypothesis of a functional overlap between the LKB1 and Notch pathways by gene expression profiling of livers deficient in *Lkb1* or in the Notch mediator RbpJκ and identified a mutual cross-talk between LKB1 and Notch signaling. *In vitro* experiments confirmed that Notch activity was deficient upon LKB1 loss.

**Conclusion:**

LKB1 and Notch share a common genetic program in the liver, and regulate bile duct morphogenesis.

## Introduction

The liver is a vital organ with many functions, one of which is bile production for lipid adsorption [1]. Bile ducts lined by cholangiocytes carry bile produced by the hepatocytes to the intestinal tract. During liver development, hepatoblasts differentiate into hepatocyte and cholangiocyte precursors which progressively mature to adult hepatocytes organized as cords and to cholangiocytes organized as ducts. Cholangiocyte precursors initially surround the portal vein mesenchyme, and form a ductal plate. The latter subsequently undergoes morphogenesis and remodelling to generate the bile ducts [2–4]. Defects in bile duct formation can impair bile duct flow eventually leading to cholestasis.

Human genetic diseases and mutant mouse models have illustrated the importance of Notch signaling in the development of bile ducts [5]. Alagille syndrome is an inherited disorder characterized by bile duct paucity and variable degree of cholestasis [6]. Nearly 80% of patients have mutations in *JAGGED1* which encodes for a Notch receptor ligand; less frequently the gene encoding for the Notch receptor NOTCH2 is mutated [7–9]. Upon ligand binding the Notch receptor undergoes sequential proteolysis releasing the intracellular domain (NICD) that translocates to the nucleus and associates with RbpJκ (Recombination signal binding protein immunoglobulin J kappa) to convert the RbpJκ corepressor complex into a coactivator complex that stimulates gene transcription [5]. Mouse studies showed that Notch signaling controls differentiation of bipotential hepatoblasts towards cholangiocytes as well as bile duct morphogenesis [10–17].

LKB1 is a tumor suppressor encoded by the *STK11* gene. It is an evolutionary conserved serine/threonine protein kinase implicated in a wide range of cellular functions including inhibition of cellular proliferation, regulation of cellular polarity and metabolism [18–20]. It is a multi-task kinase that acts upstream of AMPK (AMP-activated protein kinase) and 12 AMPK-related kinases [21]. LKB1 is a crucial regulator of apical epithelial cell polarity [19], and is able to polarize intestinal epithelial cells [19,22,23]. However, this effect of LKB1 may be cell-type specific, as deletion of LKB1 does not alter polarity of lung epithelial and pancreatic cells [24]. In the adult liver, LKB1 controls glucose and lipid metabolism [20,25,26]. I*n vitro* studies showed that LKB1 is required for hepatocyte polarization and establishment of the canalicular network [27]. Bile duct paucity was observed in mice bearing a deletion of LKB1 in the liver [28]. However, a developmental cause for the biliary defect was not investigated.

Here, we characterized the phenotype of mice in which the LKB1 gene has been specifically deleted in the hepatoblasts. Mutant mice were strongly cholestatic and lacked bile ducts. Studies at the prenatal stage showed that LKB1 is not required for differentiation of cholangiocyte progenitors and for ductal plate formation, but is required for bile duct morphogenesis by promoting the maturation of the primitive ductal structures. At the molecular level, we showed that LKB1 and Notch share a common genetic program in the liver, identifying a cross-talk between LKB1 and Notch that likely regulates biliary morphogenesis.

## Materials and Methods

### Animals

Mice carrying two floxed alleles on the exons III to VI of the *Lkb1* gene (*Stk11* ^lox/lox^) [29] were interbred with AlfP*-Cre* in which Cre is under the control of Albumin regulatory elements and α-feto-protein enhancer [30] to generate mice with LKB1 deletion in the hepatoblasts (LKB1^livemb^). Inactivation of the Notch pathway was carried out by crossing AlfP*-Cre* animals with mice carrying floxed allele of RbpJκ [31], an essential co-factor of NICD. All animal procedures were carried out according to French legal regulations and approved by an ethical committee, “Comité National de Réflexion Ethique sur l’Expérimenation Animale” under the registered number: CEEA34.CP.077.12. All mice were kept in well-controlled animal housing facilities.

### RNA extraction and RT-PCR

Total RNA was extracted from mouse tissues and cell lines with Trizol Reagent (Life technologies) according to manufacturer’s protocol. Reverse transcription was performed from 1 μg of total RNA using Transcriptor First Strand cDNA Synthesis Kit (Roche Diagnostics) and random hexamer as primers. Quantitative PCR reactions were run using the Light Cycler 480 Sybr Green I Master kit (Roche) and specific primers (Eurogentec) on a Light Cycler 480 thermocycler (Roche). Values were normalized with 18S ribosomal RNA. Primer sequences are indicated in S1 Table.

### Immunoblot analysis

Total protein extracts from mouse liver were obtained from 100–200 mg of frozen tissue that was bead-mill homogeneized in lysis buffer (50 mM Tris-HCl pH 7.4, 150 mM NaCl, 1 mM EGTA, 1 mM DTT, 0.1 mM AEBSF, 1% Triton X-100), supplemented with a mixture of protease and phosphatase inhibitors (Roche) in a 10μl/μg ratio using a TissueLyser disruption system (Qiagen, Hilder, Germany). Samples were centrifuged at 13.000 g for 10 min at 4°C and supernatant was collected and kept at -80°C until analysis. Proteins were resolved by SDS–PAGE, transferred to nitrocellulose and blocked with 5% BSA or 5% milk. Blots were incubated with specific primary antibodies overnight at 4°C, washed, incubated with the corresponding horseradish peroxidase-conjugated secondary antibodies (Cell Signaling) and developed by enhanced chemiluminescence (Thermo Fisher Scientific, Waltham, MA). Images were recorded using a super CCD camera of 3.2 megapixels driven by the LAS 4000 mini device (GE Healthcare). LKB1 antibody (clone D60C5), AMPK and anti-phospho-AMPKαT172 were from Cell Signaling Technologies. β-actin antibody was from Sigma Aldrich.

### Blood biochemistry

Bilirubin levels were measured from plasma using the Bilirubin SF kits from Diasys according to the manufacturer instructions. ALAT levels were measured from plasma using the ALATSF kit from Diasys and according to the manufacturer instructions

### Immunohistochemistry and Immunofluorescence

Mouse liver were minced in 3mm-thick sections and fixed in 10% formalin for 12 hours and embedded in paraffin. For morphological analysis, dewaxed 2μm sections were stained with hemalun and eosin.

CK19 and CD10 immunostaining procedures were performed on 5μm thick dewaxed tissue section, boiled in pH6 citrate buffer for 40 minutes and incubated for 1h at room temperature with primary antibody. After incubation with biotinylated secondary antibody, an avidin-biotin amplification step was performed (Jackson laboratories) followed by a diaminobenzidine-based revelation (Jackson laboratories) and counterstaining in hemalun. Anti-CD10 immunohistochemistry was performed using the MOM kit (Vector Laboratories). Anti-CK19 was a gift from Sylvie Germain and anti-CD10 was from Tebu-Novocastra. Immunofluorescence staining for aPKCζ was done on frozen sections. Antibody anti- aPKCζ was from Santa-Cruz.

For immunostaining of developing bile ducts, embryos were fixed at 4°C for 4h in 4% paraformaldehyde in PBS, washed overnight in PBS and embedded in paraffin. Tissue sections were retrieved by boiling for 10 min in pH6 citrate buffer, permeabilized for 15 minutes with 0.3% Triton X-100-PBS, and blocked in 3% milk/10% BSA/0.3% Triton X-100 in PBS for 45 minutes at room temperature. Primary antibodies were purchased from Santa-Cruz (HNF4, HNF6 and HNF1β), BD biosciences (E-cadherin), Chemicon (Sox9), R&D systems (osteopontin). Anti-HES1 was provided by B. Stanger, and anti-Notch2 NICD developed by S. Artavanis-Tsakonas was obtained from the Developmental Study Hybridoma Bank, created by the NICHD of the NIH and maintained at the University of Iowa, Department of Biology, Iowa City, IA 52242. Incubation of primary antibodies was performed in 3% milk/10% BSA/0.3% Triton X-100 in PBS for 1 hour at 37°C. Washes were done with 0.1% Triton X-100 in PBS three times for 5 minutes each. Secondary antibodies Alexa Fluor^®^ conjugated were purchased from LifeTechnologies. Washes were repeated with 0.1% Triton X-100-PBS three times for 5 minutes each and slides were mounted in Dako^®^ fluorescent mounting medium (Dako). Fluorescence was immediately observed with a Zeiss Axiovert 200 inverted fluorescence microscope. All the pictures were taken using a Coolpix 995 digital camera (Nikon).

### Microarray analysis, statistical analysis and data mining

Three hundred ng of total RNA were reverse transcribed following the Genechip Whole transcript (WT) Sense Target labelling assay kit (Affymetrix). The resulting cDNA was used for *in vitro* transcription with T7 RNA polymerase. After purification, 10 μg of cRNA was used for reverse transcription with random primers. The cDNA obtained was purified and fragmented. After control of fragmentation using 2100 Bioanalyzer, cDNA was end-labelled with biotin using Terminal Transferase (WT terminal labelling kit, Affymetrix). cDNA was then hybridized to GeneChip^®^ Mouse Gene (Affymetrix) at 45°C for 17 hours. Chips were washed on the fluidic station FS450 following specific protocols (Affymetrix) and scanned using the GCS3000 7G. The image was analyzed with Expression Console software (Affymetrix) to obtain raw data (cel files) and metrics for Quality Controls. Data have been deposited in GEO database (GSE75564).

Microarray data were analyzed using R-based BRB-Array Tools as previously described [32]. Briefly, differentially expressed genes were identified by a univariate two-sample t-test with a random variance model. Individual genes were selected on the basis of both statistical significance (p<0.001) and fold change (FC) difference between the compared groups (FC>1.5). False discovery rate (FDR)/q-value has been calculated as previously described [33].

Ingenuity pathway analysis (IPA) software (Mountain View, CA, USA) was used to examine the functional association between differentially expressed genes and to generate the most significantly altered molecular functions that were identified using the scoring system provided by IPA. Gene set enrichment analysis (GSEA) was performed by using the Java-tool developed at the Broad Institute (Cambridge, MA, USA).

### Cell Culture and Transfections

The human cholangiocarcinoma Mz-ChA-1 [34], a gift of Laura Fouassier (Inserm UPMC UMRS-938, Paris France) and the human hepatocellular carcinoma cell line HUH7 were cultivated in Dubelcco’s modified Eagle’s medium (DMEM, Life Technologies) supplemented with 10% fetal bovine serum and 100U/ml penicillin/streptomycin at 37°C under 5% CO_2_. For transfection, 0.5x10^6^ cells were seeded in each well of a 6-well plate. The Notch RBPJ reporter plasmid contains four copies of the RbpJκ-binding element cloned upstream of a SV40 promoter-driven luciferase reporter construct and was a kindly gift from Evelyne Lauret (Institut Cochin, Paris, France). The expression vector encoding Notch 1 intracellular domain (NICD) was a gift from Evelyne Lauret (Institut Cochin, Paris, France). The human LKB1 siRNA pool is a SMART selection designed from Thermo Fischer Scientific, Dharmacon Product. Human scrambled siRNA (ON-TARGETplus-Non-Targeting pool) was from Dharmacon. RSV-Renilla was used to normalize transfection efficiency. Cells were either co-transfected with increasing doses of NICD, 250 ng of RBPJ reporter and 10 ng of RSV-Renilla, or co-transfected with 50 or 100 pmol of siRNA, 250 ng of RBPJ reporter and 10 ng of RSV-Renilla. The transfection was done using the Lipofectamine^®^ RNAiMAX protocol according to the manufacturer’s protocol (Life Technologies). Forty-eight hours after transfection, Luciferase activity was measured with the Dual-Luciferase reporter assay system (Promega, Madison, WI). Results were expressed as firefly luciferase activity normalized to Renilla luciferase activity of the same sample. Each point was done in triplicate.

## Results

### Liver cholestasis is induced in mice with LKB1 deletion in hepatoblasts

**Fig 1:**
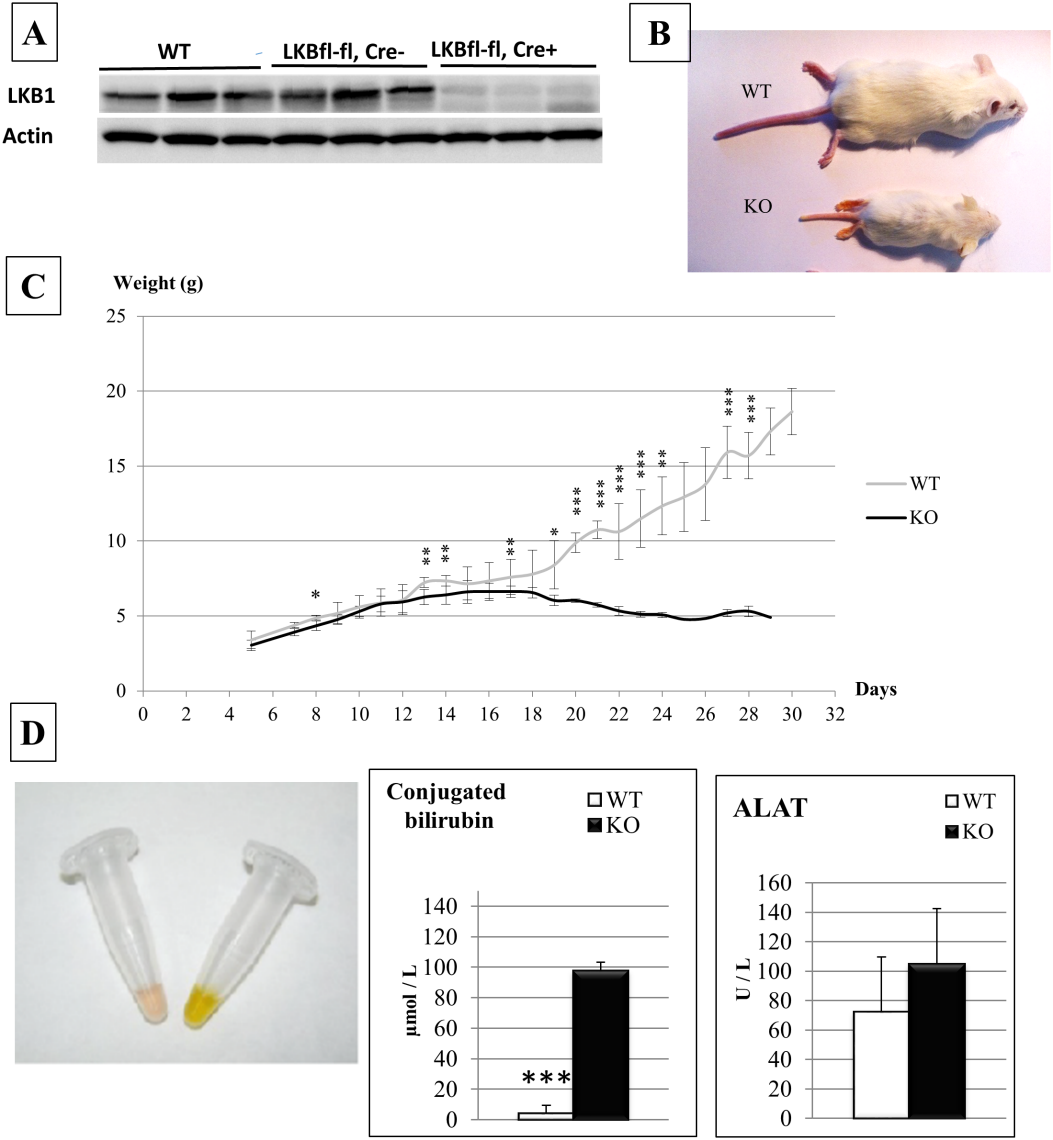
Phenotype of mice carrying *Lkb1* deletion in the embryonic liver. **(A**) LKBKO^livemb^ mouse model results in efficient inactivation of Lkb1 protein expression in the liver. Western blot analysis of Lkb1 and β-actin (loading control) of liver lysates from wild-type, control (LKB1^fl,fl^, Cre-) designed WT and mutant (LKB1^fl,fl^, Cre+) mice designed KO (2-week old mice). (B) Gross appearance of a control (WT) and mutant (KO) LKBKO^livemb^ mice at postnatal day 28. (**C)** Weight curves from birth to post-natal day 30 in the LKBKO^livemb^ model. Mice genotypes were determined at postnatal day 5. N = 15 control and 8 mutant mice. Error bars: standard deviations.* P<0.01, ** P<0.05, *** P<0.001. Deletion of *Lkb1* in the embryonic liver causes postnatal growth retardation beginning at day 12. (**D)** Obstructive cholestasis in LKBKO^livemb^ mutant mice. Gross aspect of serum from a control (WT) and mutant (KO) LKBKO^livemb^ mice at postnatal day 15. Blood levels of conjugated bilirubin and ALAT in LKBKO^livemb^ control (WT) and mutant (KO) mice at postnatal day 15. n = 6 control and 4 mutant mice. Error bars: standard deviations. Statistical significance was evaluated using a two-sample unpaired Student’s t-test between KO and mutant animals. *** P<0.001.

We specifically deleted *Lkb1* in the liver using mice with floxed *Lkb1* ^*lox/lox*^ alleles [29] and transgenic *Alfp-Cre* [30]. In the latter, Cre recombinase is active in hepatoblasts starting at E10.5. The mutant animals were named LKBKO^Livemb^. After confirming that *Lkb1* was efficiently deleted in the liver (Fig 1A), we studied the phenotype of these mice. LKBKO^Livemb^ mice were born at the expected Mendelian frequency, but showed severe growth retardation beginning at post-natal day 12 and leading to death between day 25 and day 30 (Fig 1B and 1C). The severity of the phenotype of LKBKO^Livemb^ mice differed from that observed in the work by Woods et al. [28]. In the latter, defective growth was detected at an earlier stage (post-natal day 4) while we observed growth defect at the suckling/weaning transition. Furthermore, Woods et al. detected cytolysis as assessed by elevated transaminase levels; in contrast, we found normal serum ALAT levels in LKBKO^Livemb^ mice. The difference in the liver phenotype between the two studies is likely explained by the genetic difference of the two models: Woods’ study was done using an hypomorphic floxed Lkb1 model harbouring strong reduction in LKB1 protein expression in Cre-negative mice [35] while no such decrease was observed in our Cre-negative mice (Fig 1A).

**Fig 2:**
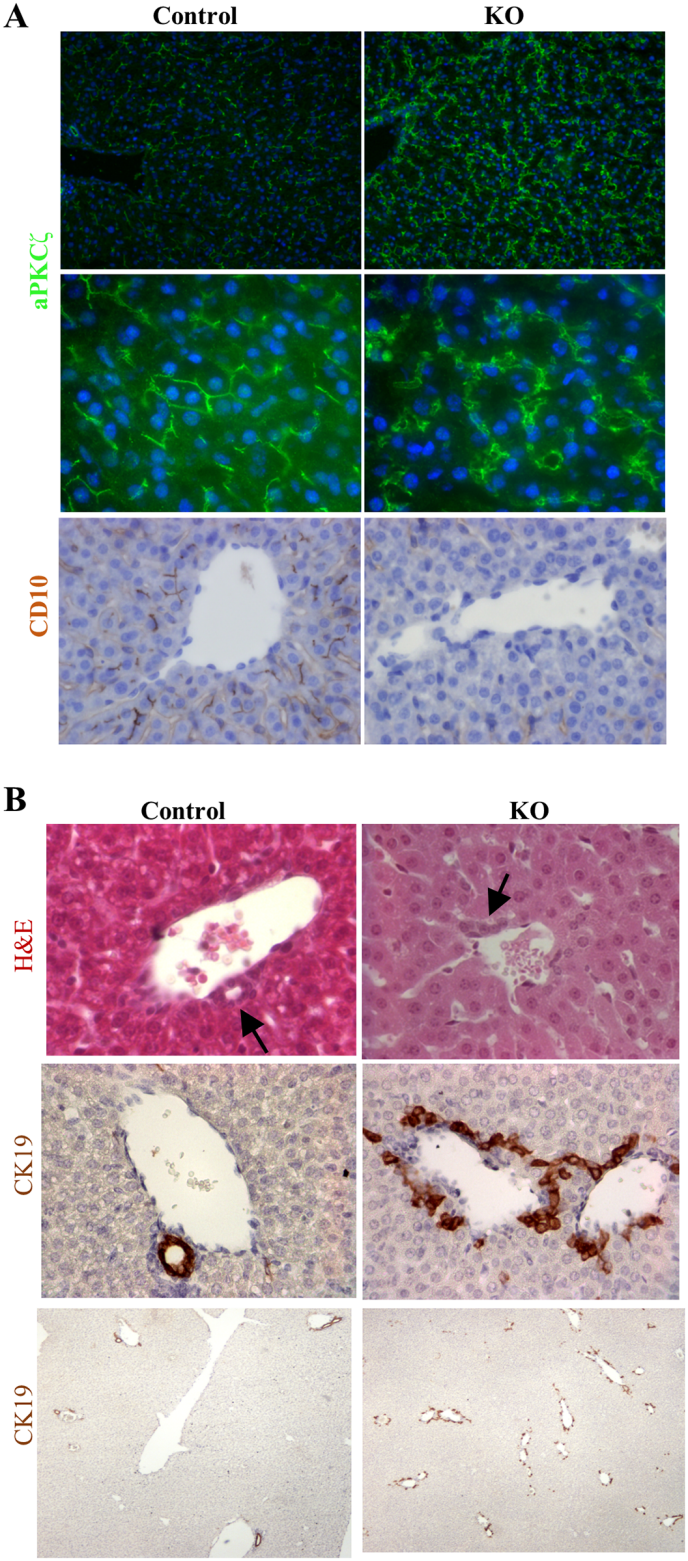
Lkb1 is required for canaliculi formation and intrahepatic bile ducts morphogenesis. (A) Top panel: representative images of 3-week old control and mutant LKBKO^livemb^ mice liver sections stained with the apical marker aPKCζ. Note the elongated canalicular network and the tortuous dilated bile canaliculi in control and mutant animal, respectively. Bottom panel: representative images of 3-week old control and mutant LKBKO^livemb^ mice liver sections stained with the anti-CD10 antibody. Immunohistochemistry evidences a delicate canalicular network at the apical pole of the hepatocytes of control animal. In KO mice, the staining was lost. Top: low magnification, middle and bottom: high magnification. (B) Top panel: Hematoxylin-eosin (H&E) stained sections of 3-week old control and mutant LKBKO^livemb^ mouse liver. Middle and bottom panels: Cytokeratin 19 (CK19)-stained sections of 3-week old control and mutant LKBKO^livemb^ mouse livers. Note the well-formed and mature bile ducts in the control mouse and the numerous ductal plate-like structures around the portal tract in mutant mice. Top two panels: high magnification, bottom panel: scanning magnification.

The LKBKO^Livemb^ mice were strongly cholestatic as shown by the yellow color of the serum and elevated serum bilirubin levels (Fig 1D). The high level of conjugated bilirubin and the normal ALAT levels indicated that cholestasis resulted from a biliary obstruction (Fig 1D). Bile canaliculi are channels formed by the juxtaposition of apical pole of adjacent hepatocytes. Using the apical marker aPKCζ to detect the bile canaliculi, we found, as expected, an elongated and bar-shaped bile canalicular network in control animals. In contrast, the aPKCζ staining was tortuous and dilated in LKBKO^Livemb^ mice (Fig 2A). Mutant mice failed to express the hepatocyte canalicular membrane, CD10 [36], reinforcing that bile canalicular network was defective (Fig 2A). Similar results were observed in the Wood’s study [28]. We then examined the intrahepatic bile ducts in LKBKO^Livemb^ mice. In control livers at postnatal day 15, one to two CK19-positive bile ducts were located in each portal tract (Fig 2B). In contrast, mutant animals showed CK19-positive cells organized around the portal tract like embryonic ductal plate structures, but failed to develop ducts (Fig 2B). Thus, LKBKO^Livemb^ mice revealed defects in hepatocytes with aberrant apical polarization leading to defective bile canaliculi, and defects in bile duct formation that all were responsible for the cholestasis.

### LKB1 promotes maturation of bile ducts during biliary morphogenesis

To determine how LKB1 deletion causes defective duct formation, we analyzed bile duct morphogenesis at prenatal stages. During normal embryonic development, hepatic progenitor cells adjacent to the portal vein form a structure composed of cholangiocyte precursors, called ductal plate. Then, prior to birth, tubulogenesis occurs at discrete areas along the ductal plate, giving rise to asymmetrical ducts, called primitive ductal structures (PDS) lined by Sox9+/HNF4- cholangiocytes and Sox9-/HNF4+ hepatoblast-like cells. Around embryonic day E17-E18, these structures mature to bile ducts entirely and symmetrically lined by Sox9+/HNF4- cholangiocytes [3,37]. Cholangiocyte precursors of the ductal plate not involved in duct formation give rise to periportal hepatocytes and to cells of the Hering’s canal [38].

**Fig 3:**
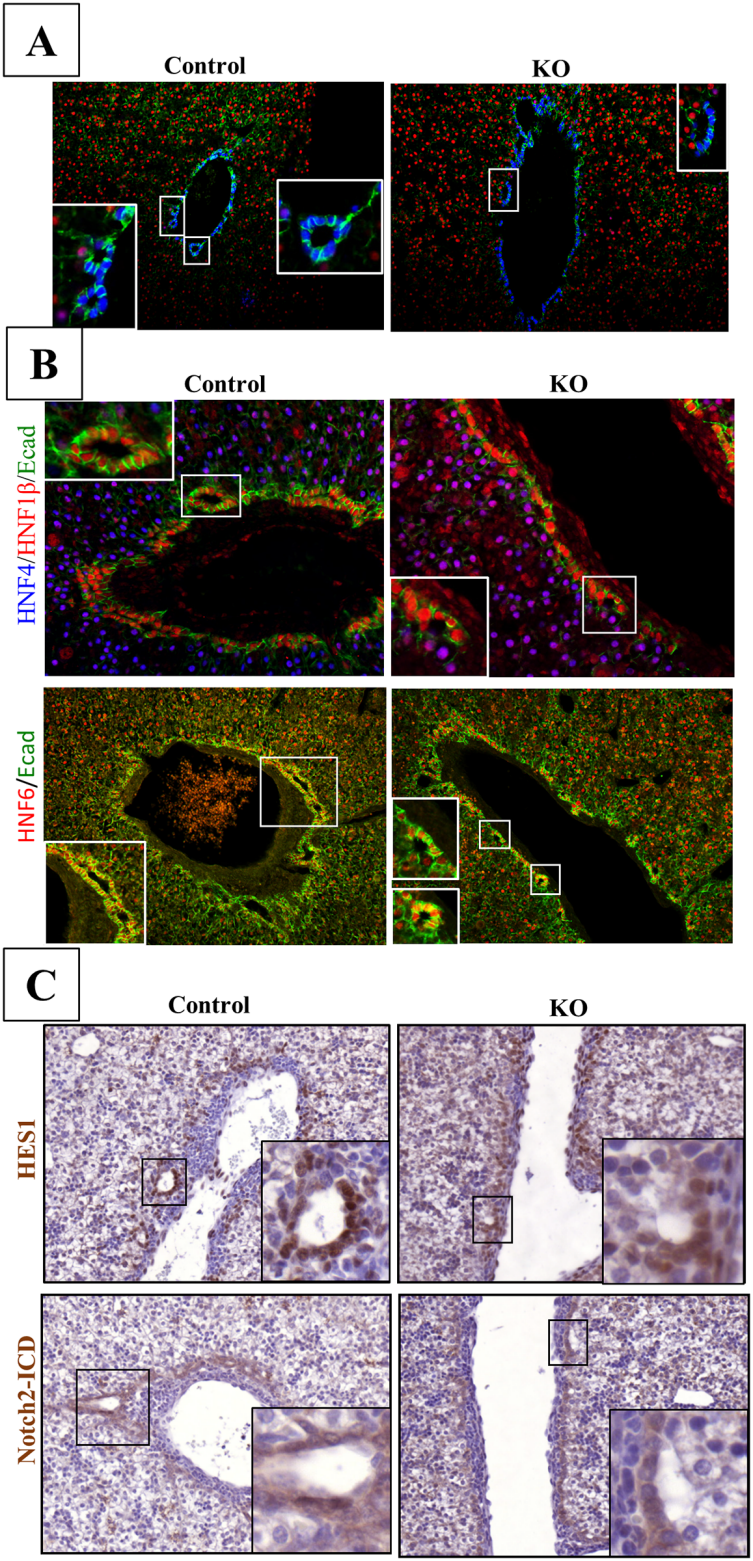
Lkb1 controls the maturation of bile duct during bile duct tubulogenesis. (A) Immunofluorescence for Sox9, HNF4 and E-cadherin demonstrate that Lkb1 is required for the transition from an asymmetric primitive duct to a symmetric and mature bile duct in the developing liver. LKBKO^livemb^ (KO) embryos were sampled at E18.5 and liver sections were stained for the hepatoblast marker HNF4, and for the cholangiocyte markers Sox9. Note the symmetrical localization of Sox9 around the bile duct in control mice (Control) whereas Sox9 was only expressed in the portal layer of the asymmetric bile ducts in mutant embryos. High E-cadherin levels mark mature cholangiocytes. (B) Representative immunofluorescence for HNF1β and HNF6 in control and LKBKO^livemb^ livers in top and bottom panels respectively. Medium-high magnifications. (C) Notch-ICD and Hes-1 expression are restricted to the portal layer of asymmetrical bile duct in LKBKO^livemb^ mutant mice whereas Notch activation was evidenced in both layers of the biliary tubules in control animals. Medium magnification.

In control animals, at E18.5, developing ducts were symmetrical and completely lined with Sox9+/HNF4- cells. In contrast, at that stage developing ducts of LKBKO^Livemb^ mice were still in an asymmetrical configuration typical of PDS: the duct lumina were surrounded on the portal side by Sox9+/HNF4- cholangiocytes and on the opposite, *i*.*e*. parenchymal side, by Sox9-/HNF4+ hepatoblasts (Fig 3A). We then tested the expression of key transcription factors of bile duct morphogenesis, namely HNF1β and HNF6 [39–41]. These factors were normally expressed on the parenchymal and portal sides of developing ducts, in wild-type livers and only in the portal side of the ducts of the LKBKO^Livemb^ livers (Fig 3B). As well, the nuclear location of the Notch intracellular domain (NICD) and the expression of the Notch target gene Hes1 were asymmetrical in developing ducts of mutant embryos, in contrast to control animals revealing a symmetrical expression (Fig 3C). These data demonstrated that in the absence of LKB1, duct morphogenesis is arrested at the PDS stage and that Notch signaling is not functional on the parenchymal side of the developing ducts in the absence of LKB1. In mutant animals, the PDS do not mature to bile ducts and, together with the other cholangiocyte precursors not involved in duct formation, remain at an embryonic stage, explaining the ductal-plate-like structure observed in adult animals.

### Cross-talk between LKB1 and Notch pathways in liver

**Fig 4:**
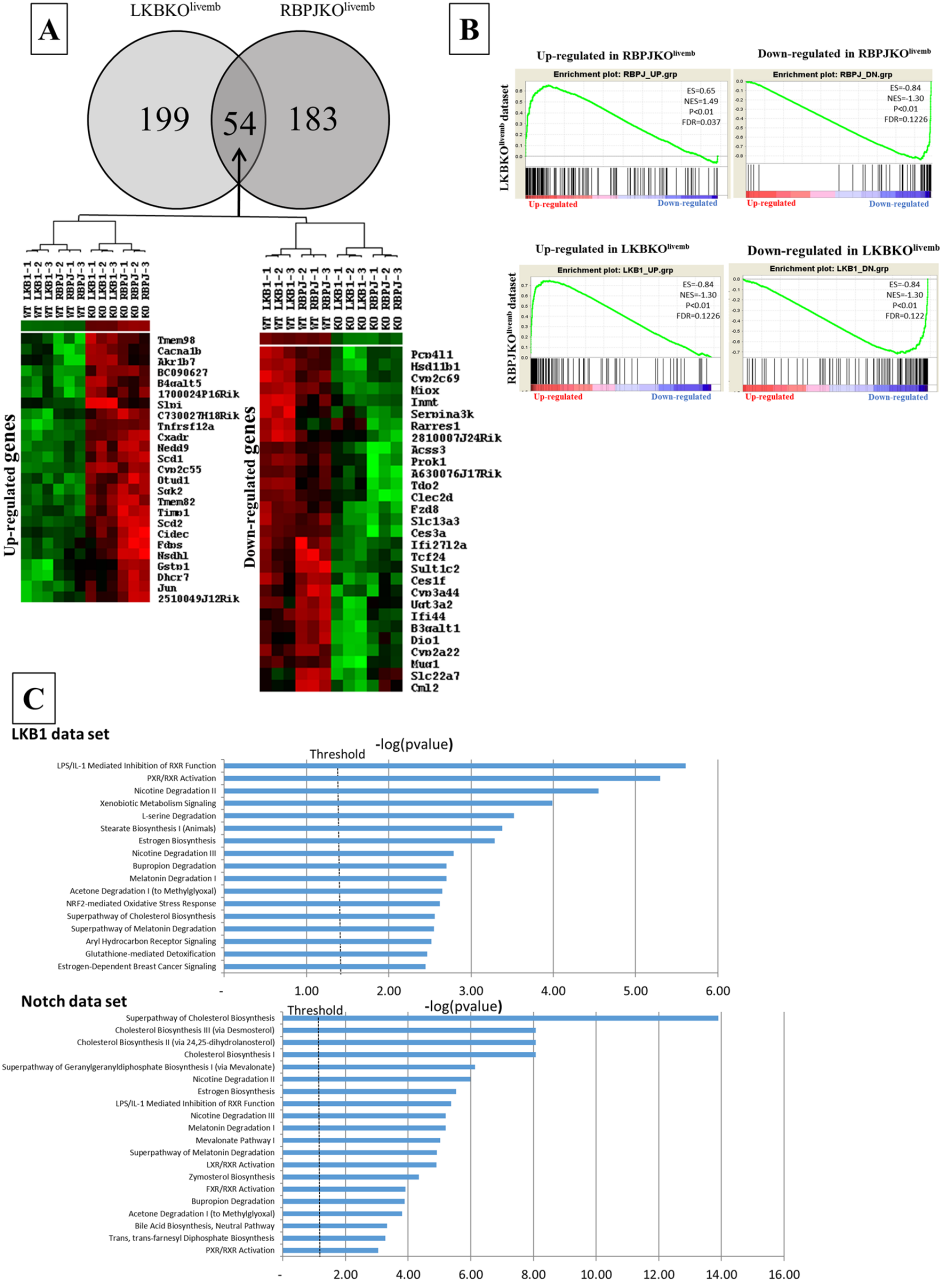
Inactivation of *Lkb1* and Notch in the liver share a common gene signature. (A) RBPJKO^livemb^ and LKBKO^livemb^ models share a common gene expression signature. Upper part: Venn diagram of genes differentially expressed (p<0.001, fold change>1.5) between mutant and control mice in the LKBKO^livemb^ and RBPJKO^livemb^ (5-days old) models. Fifty-five genes were found to be deregulated in the two models. Lower part: Supervised hierarchical clustering analysis demonstrates that 54 (out of 55) common genes are similarly deregulated in the two models. (B) Gene Set Enrichment Analysis (GSEA) demonstrates that the Lkb1 and Notch pathways share a common transcriptional program. RBPJKO^livemb^ (upper part) and LKBKO^livemb^ (lower part) gene signatures were used for GSEA using the gene expression profiles of LKBKO^livemb^ (upper part) and RBPJKO^livemb^ (lower part) mice and their respective control (WT) counterparts. Up- and down-regulated genes in the RBPJKO^livemb^ signature were found to be specifically enriched in the gene expression profiles of LKBKO^livemb^ and control (WT) mice, respectively. Similarly, up- and down-regulated genes in the LKBKO^livemb^ signature were found to be specifically enriched in the gene expression profiles of RBPJKO^livemb^ and control (WT) mice, respectively. All gene sets were significantly enriched at nominal p-value<1%. (C) Most significantly altered functions revealed by Ingenuity Pathway Analysis (IPA). A dataset containing gene identifiers and corresponding values were uploaded to the Ingeniuty Pathway analysis software (IPA). The transcripts differentially expressed between KO and WT that met the cutoff criteria (FC > 1.5, p< 0.001) were considered for the analysis. Bars represent the logarithmic value of the significance level, the dashed line corresponds to the threshold of 0.05.

The biliary phenotype of LKBKO^Livemb^ mice is reminiscent of that observed in mice with inactivation of Hes1, Jagged 1, or RbpJκ [11,15,17]: all these mice develop a ductal plate which fails to generate ducts. This suggested that LKB1 cross-talks with the Notch pathway in the liver. To check this hypothesis we analyzed *Rbpj* ^*lox/lox*^;*Alfp-Cre* (RBPJKO^livemb^) mice which have a liver-specific inactivation of RbpJκ. Control, RBPJKO^livemb^ and LKBKO^Livemb^ mice were sacrificed 5 days after birth and the liver transcriptomes were analyzed using microarrays. 253 non redundant genes were differentially expressed between LKBKO^Livemb^ and control mice (see S2 Table); 237 genes were differentially expressed between RBPJKO^livemb^ and control mice (see S3 Table). Interestingly, numerous genes (54 out of 55 genes, *i*.*e*. 20–25% of each list, see S4 Table) were similarly deregulated in the liver of LKBKO^Livemb^ and RBPJKO^livemb^ mice (Fig 4A). Accordingly, Gene Set Enrichement Analysis (GSEA) revealed that the LKBKO^livemb^ gene signature was significantly enriched in the gene expression profile of RBPJKO^livemb^ livers, and *vice versa* (Fig 4B). Analysis of functional gene networks using Ingenuity Pathway Analysis highlighted the metabolic pathways as the main deregulated pathways in both the LKB1 and Notch gene datasets (Fig 4C).

**Fig 5:**
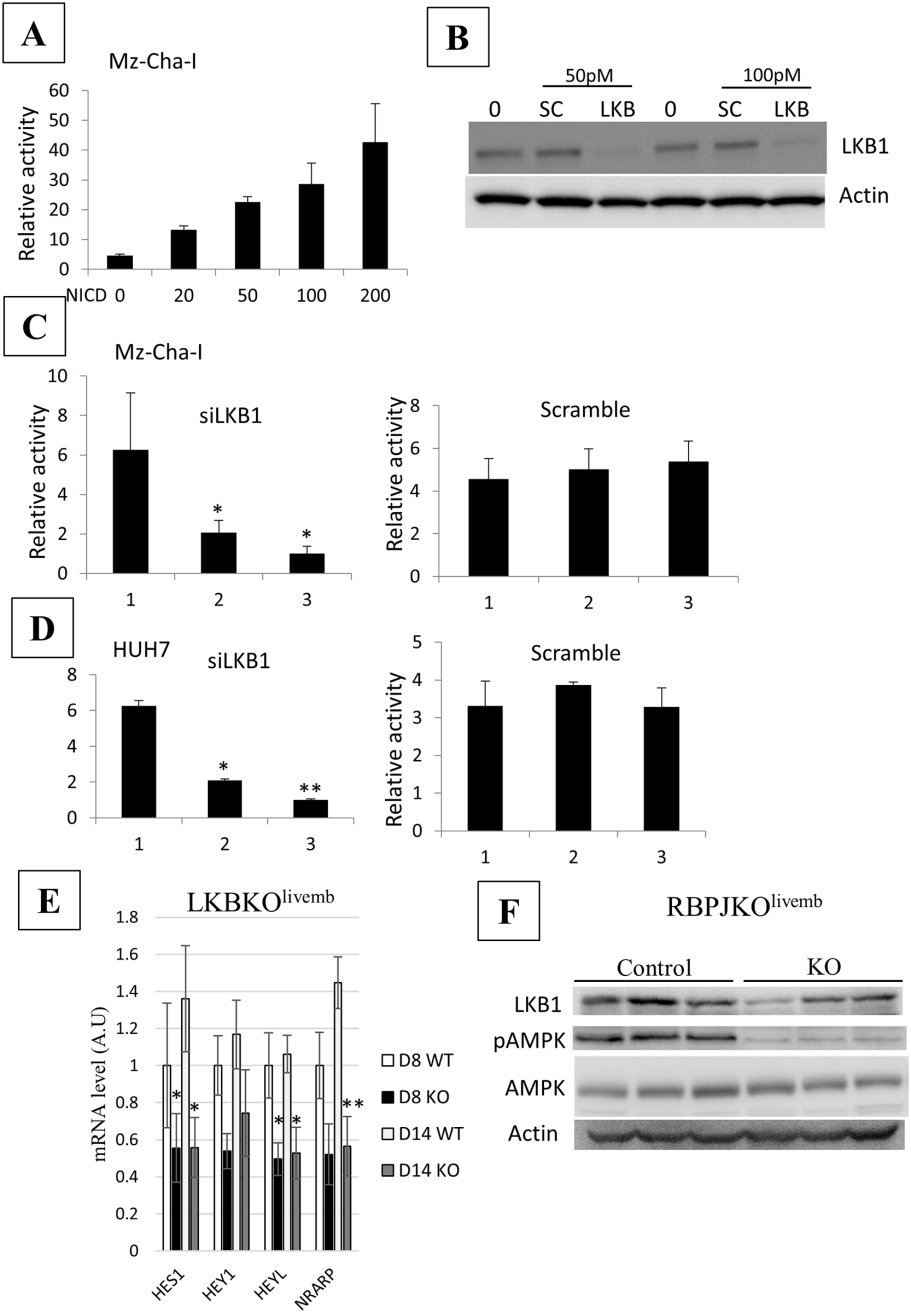
Cross-talk between LKB1 and Notch signaling. **(A**) The RBP-J luciferase reporter was transfected into Mz-Cha-1 cells either alone (0) or with increasing dose of NICD vector (ng) as indicated. (B) Silencing by 50 pmol -100 pmol of LKB1 siRNA (LKB) and scrambled siRNA (SC). LKB1 protein was revealed by western blot. β-actin was used as loading control. (C-D) LKB1 silencing led to decrease Notch activity. Luciferase activity was measured in the absence of siRNA (1), in the presence of either 50 pmol of LKB1 siRNA or scrambled siRNA (2) and the presence of 100 pmol of either LKB1 siRNA or scrambled siRNA (3). Transfections were done in Mz-ChA-1 cells (C) or in HUH7 cells (D). * P< 0.05. ** P<0.01. (E) LKB1 is required for full activation of Notch signaling in the developing liver. RT-qPCR of different Notch positive target genes (*Hey1*, *Heyl*, *Hes1* and *Nrarp*) in 8- and 14-day old control and mutant LKBKO^livemb^ mice. N = 3–4 per group. Error bars: SEM. **F**: Notch negatively regulates LKB1 level measured by western blot analysis and the phosphorylation level of AMPK (pAMPK-T172). Statistical significance was evaluated using a two-sample unpaired Student’s t-test between KO and WT. * P< 0.05. ** P<0.01.

To confirm *in vitro*, the cross-talk between LKB1 and Notch signaling pathways the cholangiocarcinoma cell line Mz-ChA-I was used. A Notch-responsive luciferase reporter system containing RbpJκ binding sites [42] allowed us to monitor the level of Notch activation. Transfection of NICD induced a dose-dependent increase in luciferase activity, as expected (Fig 5A). More interestingly, we observed a decrease in luciferase activity after LKB1 inactivation by siRNA and not in cells transfected with a scrambled siRNA (Fig 5B and 5C). This result confirmed that Notch activation is deficient in the absence of LKB1. Similar results were obtained using the hepatocellular carcinoma cell line HUH7 (Fig 5D), indicating that the dialog between LKB1 and Notch signaling extends beyond bile duct cells. We then search for an epistatic relation between the LKB1 and Notch signaling, and characterized the activation status of the Notch pathway in LKB1 mutant animals, and reciprocally, the level of LKB1 expression in Notch mutant livers. Expression of several Notch pathway targets (*Hes1*, *Hey1*, *Heyl* and *Nrarp*) was downregulated in the liver of LKBKO^Livemb^ mutant mice corroborating the results we obtained using the Mz-ChA-I cell line (Fig 5E). Reciprocally, Notch inactivation was associated with down-regulation of LKB1 protein and its downstream kinase, AMPK, as revealed by the level of phosphorylated AMPK (Fig 5F). These results showed a mutual cross-regulation between the LKB1 and Notch pathways without evidence that one is epistatic to the other.

## Discussion

In the present work we identify a new role of LKB1 in bile duct development. In the absence of LKB1 differentiation of hepatoblasts to cholangiocyte precusors proceeds normally; the precursors organize as a ductal plate and generate PDS, but the latter fail to mature to ducts. A similar maturation defect has been observed in the absence of HNF1β [41]. We showed that Notch pathway activation was deficient in the absence of LKB1, by *in vivo* and *in vitro* approaches. Since Notch pathway deficiencies are associated with a biliary phenotype similar to that of LKBKO^Livemb^ mice, we suggest that LKB1 controls Notch signaling during bile duct development.

The failure of PDS to mature and generate ducts may reflect polarity defects in LKBKO^Livemb^ mice. When PDS mature to ducts, the cells on the parenchymal side become progressively more polarized and expressed higher level of E-cadherin [37]. A co-localization between LKB1 and E-cadherin has been described at the adherens junction and E-cadherin is required for the recruitment of active LKB1 complex to adherens junction in polarized epithelial cells [43]. Thus, a polarity clue mediated by LKB1 may be the signal that allows the transition of PDS to a symmetric bile duct. In addition, deficient apical polarization of hepatocytes was likely to explain the abnormal development of bile canaliculi in mutant mice. Therefore, polarization of hepatocytes and cholangiocytes may constitute a key function of LKB1 during liver development.

Hepatocytes have a marked anatomical polarity that plays an essential role for biliary secretion. Several bile acid transporters are localized at the canalicular apical pole. *In vitro* studies showed that formation and maintenance of bile canalicular network of the hepatocytes is regulated by the LKB1 and AMPK pathways [27]. Accordingly, we observed loss of the bile canalicular network in LKBKO^Livemb^ mice. Thus alterations in both hepatocytes and cholangiocytes explain the cholestatic phenotype of mutant mice which do not have mature bile ducts and present defective bile canalicular network.

At the molecular level, our *in vivo* and *in vitro* analyses showed that LKB1 loss led to a decrease in Notch activity, but the mechanism by which LKB1 controls Notch activation remains to be investigated. Interestingly, our results indicated that Notch-deficient mice displayed a decrease in the LKB1 activity highlighting a cross-regulation of LKB1 and Notch signaling. The liver is not the only organ in which LKB1 and Notch cross-talks. A connection between the LKB1 and Notch pathway has been recently described in the intestinal epithelium. Deletion of LKB1 in the epithelial cells of the intestine is associated with a modification of the differentiation of the intestinal lineage towards an increase in goblet and Paneth cell lineage known to be negatively controlled by Notch signaling [44]. Similar to our results, a decrease in Notch activation was evidenced in intestine of mice bearing specific deletion of LKB1 [45]. Therefore, our work and that of others point toward a combinatorial role of LKB1 and Notch in cell fate decision and organ morphogenesis.

Our gene expression profiling results further highlight that the Notch pathway has roles that extend beyond development and that it impacts on organ homeostasis [5]. Recent data show that Notch participates in liver glucose and lipid homeostasis [46,47]. Accordingly numerous genes linked to lipid metabolism were present in the shared LKB1-Notch dataset (see Fig 4A).

## Conclusion

Liver-specific deletion of LKB1 in transgenic mice identified LKB1 as an actor of bile duct maturation during biliary morphogenesis. Our data suggest that a mutual cross-talk between LKB1 and the Notch pathway is involved in bile duct morphogenesis.

## Supporting Information

S1 Table List of primer sequences. (DOCX)

S2 Table List of 253 non-redundant genes differentially expressed in KO vs WT LKB1 mice. * p < .001; ** fold-change >1.5.
(XLSX)

S3 Table List of 237 non-redundant genes differentially expressed in KO vs WT RBPJ mice. * p < .001; ** fold-change >1.5.
(XLSX)

S4 Table List of 54 non-redundant genes differentially expressed in both LKB1 and RBPJ KO vs WT mice. * p < .001; ** fold-change >1.5.
(XLSX)

## Abbreviations

PDS: primitive ductal structure
RbpJκ: Recombination signal binding protein immunoglobulin J kappa
