# Supplementary material for: LKB1 and Notch Pathways Interact and Control Biliary Morphogenesis
Source: PLoS One. 2015 Dec 21;10(12):e0145400. doi: 10.1371/journal.pone.0145400 (PMC4687046; doi:10.1371/journal.pone.0145400)
Supplement: S1 Table — (DOCX) [file pone.0145400.s001.docx]

S1Table : List of primers sequences

| Species | Gene |  | Sequence |
| --- | --- | --- | --- |
| Mouse | *Hes1* | Fwd | tgccagctgatataatggagaa |
|  |  | Rv | ccatgataggctttgatgacttt |
| Mouse | *Hey1* | Fwd | catgaagagagctcacccaga |
|  |  | Rv | cgccgaactcaagtttcc |
| Mouse | *Heyl* | Fwd | catgaagagagctcacccaga |
|  |  | Rv | cgccgaactcaagtttcc |
| Mouse | *Nrarp* | Fwd | gctacacatcgccgcttt |
|  |  | Rv | ttggccttggtgatgagata |
